# Correlation Analysis and Verification of Railway Crossing Condition Monitoring

**DOI:** 10.3390/s19194175

**Published:** 2019-09-26

**Authors:** X. Liu, V. L. Markine

**Affiliations:** Section of Railway Engineering, Delft University of Technology, 2628 CN Delft, The Netherlands

**Keywords:** railway crossing, condition monitoring, condition indicator, correlation analysis, weather impact, numerical verification

## Abstract

This paper presents a correlation analysis of the structural dynamic responses and weather conditions of a railway crossing. Prior to that, the condition monitoring of the crossing as well as the indicators for crossing condition assessment are briefly introduced. In the correlation analysis, strong correlations are found between acceleration responses with irregular contact ratios and the fatigue area. The correlation results between the dynamic responses and weather variables indicate the influence of weather on the performance of the crossing, which is verified using a numerical vehicle-crossing model developed using the multi-body system (MBS) method. The combined correlation and simulation results also indicate degraded track conditions of the monitored crossing. In the condition monitoring of railway crossings, the findings of this study can be applied to data measurement simplification and regression, as well as to assessing the conditions of railway crossings.

## 1. Introduction

**Figure 1:**
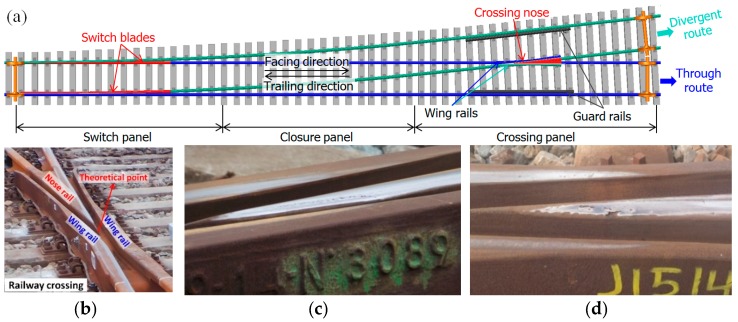
(**a**) Standard left-hand railway turnout with a 1:9 crossing (drawn by X. Liu); (**b**) crossing panel on site (shot by V.L. Markine); (**c**) plastic deformation with cracks (shot by X. Liu); (**d**) spalling (shot by X. Liu).

Railway turnouts are essential components of railway infrastructure and provide the ability for trains to transfer from one track to the other. In the meantime, a gap between the wing rail and nose rail in the crossing panel (Figure 1b) introduces a discontinuity in the rail. As a result of trains passing through, the high wheel–rail impact due to the high train velocity causes this type of crossing to suffer from severe damage such as cracks (Figure 1c) and spalling (Figure 1d), and the service lives of some railway turnouts are only 2–3 years. In order to improve the maintenance of the crossing and prolong service life, it is better to perform maintenance in a predictive way by developing a structural health monitoring (SHM) system for railway crossings [1].

In order to obtain information on damage detection, localization and condition assessment in SHM systems, it is important to get insight into the performance of the structures. In recent years, SHM has drawn increasingly more attention in the railway industry. D. Barke and W.K. Chiu reviewed the major contributions of condition monitoring in regards to wheels and bearings [2]. Based on digital image correlations, D. Bowness et al. measured railway track displacement using a high speed camera [3]. The axle box acceleration (ABA) system has been widely applied in the condition monitoring [4] and damage detection [5,6] of railway tracks. However, most of the contributions of SHM are based mainly on normal tracks. Z. Wei et al. have applied the ABA system in railway crossing damage detection [7]. However, as a special and vulnerable component in the railway track system, the study on crossings in terms of condition monitoring are still limited. 

In the existing studies, the performance analysis of crossing has been based mainly on numerical approaches. For instance, finite element (FE) wheel-crossing models have been applied to calculate plastic deformation and frictional work [8], to simulate the distribution of stresses in the crossing nose [9] and to predict the fatigue life of a crossing [10]. Also, multi-body system (MBS) vehicle-crossing models have been used for general train–track interaction analysis [11], track elasticity analysis [12], crossing geometry optimization [13,14,15] and so on. Due to restricted track access, high costs and time consumption, field measurements have mainly been used for numerical model validation [9,16]. The numerical models are usually developed according to a certain hypothesis with a focus on specific problems. However, for damage detection and assessments of crossing conditions, the numerical approach alone is not enough, and monitoring the conditions of in-service railway crossings is highly necessary.

In real life, the wheel–rail contact in a crossing can be affected by many factors. Some factors are related to the train track system, such as train type [17], velocity [18], axle load [18,19], wheel–rail friction [18], crossing geometry [18,19], track alignment [19], track elasticity [12] and so on. Some factors are related to the crossing environment, such as the contaminants on the rail [19,20,21] and rail temperature variation [22,23]. All these factors, especially those introduced by the environment, make the measurement data noisy and the crossing condition cannot be clearly shown [24]. In order to properly analyze the measurement data for monitoring the crossing condition, the first step is to figure out the influence of the above mentioned factors on the performance of the crossing.

In this study, the influence of train track system-related factors is minimized through data selection and a filtering process. Specifically, train type, velocity and the bogie number are restricted to a certain scope. In order to estimate the influence level of the external factors (such as the weather condition), a correlation analysis using Pearson’s correlation coefficient, which is usually applied to quantitatively evaluate the correlation strength between two variables, is performed. The correlation analysis results are verified using a vehicle-crossing model developed using the multi-body system (MBS) method. In this model, the weather changes are modelled according to changes in the properties of the affected track elements. The correlation between the weather condition and the dynamic responses of the crossing provides the foundation for long-term measurement data regression, which will be applied in the crossing degradation assessment procedure. In addition to weather factors, the correlation strengths between the dynamic responses of the crossing are also analyzed, which can be applied to provide guidance for the selection and post-processing of the measurement data and to improve the efficiency of analyzing a large amount of data.

The paper is organized as follows. The condition-monitoring procedure of a railway crossing, including the crossing instrumentation, is presented in Section 2. The indicators applied for the crossing condition assessment are briefly introduced in Section 3. The correlation analysis, including the dynamic responses and weather variables, are given in Section 4. In Section 5, the mechanisms of the weather effects are analyzed and verified through numerical simulation. Finally, in Section 6, the conclusions based on the correlation analysis are provided and further applications for the degradation procedure description of the monitored crossing are discussed.

## 2. Railway Crossing Condition Monitoring

In this section, monitoring the condition of a railway crossing is discussed. The crossing instrumentation and a brief procedure for processing the measurement data are described.

### 2.1. Crossing Instrumentation

**Figure 2:**
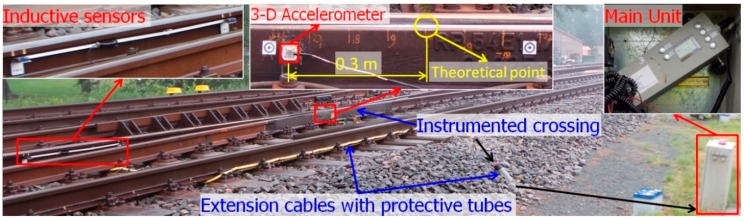
Overview of the crossing instrumentation.

The monitored crossing in this study is a cast manganese steel crossing with an angle of 1:9, which is the most commonly used crossing for Dutch railway tracks (more than 60% [25]). As part of a double crossover, the crossing is mainly used for through-facing routes (Figure 1a). This railway line is mainly used for passenger transportation with a velocity of passing trains up to 140 km/h. The crossing is instrumental for using the system that has been introduced, and has been actively used in previous studies [1,17,19,26]. An overview of the crossing instrumentation is given in Figure 2.

The main components of this device are a 3-D accelerometer attached to the crossing rail, a pair of inductive sensors attached to the rails in the closure panel and the data logger (main unit) installed on the outside of the track. The inductive sensors are used for train detection and the initiation of the measurements, as well as for train velocity determination. All of the sensors are connected to the data logger for data storage and basic analysis of the data. The measurement range and sampling frequency of the acceleration sensor are 500 g and 10 kHz, respectively. The main measured data are the 3-D acceleration responses (i.e., $a_{x}$, $a_{y}$ and $a_{z}$)) of the crossing due to the passing trains.

**Figure 3:**
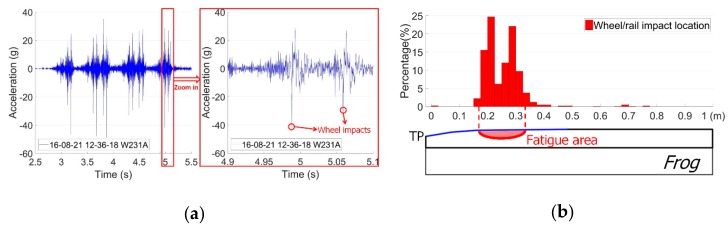
Examples of the output of crossing instrumentation. (**a**) Vertical acceleration response due to one train’s passage; (**b**) wheel impact location distribution.

An example of the vertical acceleration response in a time domain due to one passing train with 12 wheelsets is shown in Figure 3a. It can be seen from this figure that the time and location of each wheel’s impact on the crossing can easily be obtained from the acceleration responses. The region where most of the wheel impact is located is defined as the fatigue area (Figure 3b), which can be used for assessing crossing conditions based on a large amount of data.

### 2.2. Measurement Data Selection and Processing

The crossing monitored in this study was in a new state at the beginning of the observations. In order to reduce the influence of vehicle variations, the measurement results considered here were restricted to one type of train, namely the VIRM (double-deck) trains that pass with a velocity of around 140 km/h. Moreover, the accelerations caused only by the first bogie were considered. Thus, the remaining uncertainties in the measured data mainly coming from the environment (e.g., the weather). Depending on the amount of monitoring data, the measurement results will be analyzed on three different levels, namely,

- the dynamic response due to the passage of a single wheel;
- the results of multiple-wheel passages from one monitoring day; and
- the statistical results from multiple monitoring days.

**Figure 4:**
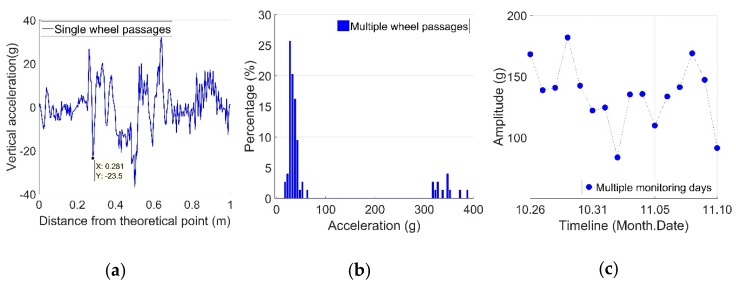
Example of measured vertical acceleration responses: (**a**) from single-wheel passages; (**b**) from multiple-wheel passages from one monitoring day; (**c**) from multiple monitoring days.

An example of vertical acceleration responses in different levels is shown in Figure 4.

The response due to single wheel passages was directly obtained from the measured time domain signal (Figure 4a). The distribution of the maximum impact acceleration from each passing wheel constituted the results of multiple wheel passages (Figure 4b). For the statistical results from multiple monitoring days, each point represented the average value of the impact vertical accelerations of the recorded passing wheels from one monitoring day (Figure 4c). It can be seen that each wheel passed the railway crossing differently. Based on a single wheel’s passage it is difficult to assess the performance of the crossing. Yet, some conclusions on wheel–rail interaction can still be drawn based on these data. The statistical analysis based on multiple passing wheels was more applicable for assessing the condition of the railway crossing.

## 3. Condition Indicators

In this section, the indicators for assessing a crossing’s condition are briefly described. These indicators are calculated based on the transition region and consist of the irregular contact ratio, 3-D acceleration responses and the fatigue area. To demonstrate the condition analysis procedure, some typical examples of the measurement results from the monitored crossing are presented.

### 3.1. Transition Region

**Figure 5:**
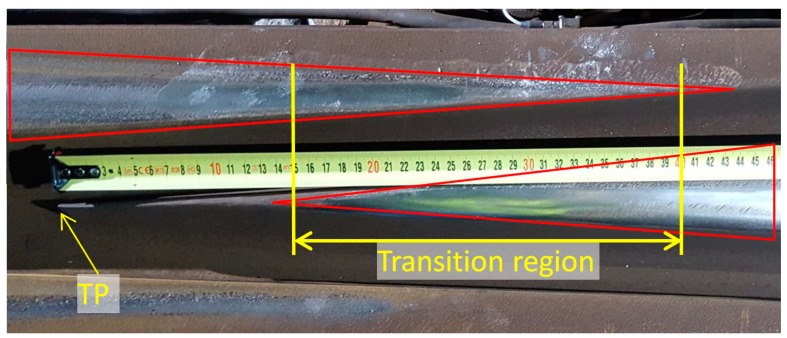
Transition region of the monitored crossing.

The transition region of a crossing is the location where the wheel load is transferred from the wing rail to the nose rail (or vice versa, depending on the traveling direction). In practice, the wheel–rail contact points in the crossing can be recognized by looking at the shining band on the rail surface. An example of such a band on the monitored crossing is given in Figure 5 and denoted by the red triangle areas. Using these bands, the transition region can be then estimated by the overlapping area of the shining bands on the wing rail and nose rail. Based on this image, the transition region of the monitored crossing is located around 0.15–0.40 m as measured from the crossing’s theoretical point (TP).

From a performance point of view, the transition region is the most vulnerable part of the crossing, since the rail is thinner and the wheel forces are higher than in the other parts of the turnout. Therefore, to analyze the dynamic performance of the crossing, only the accelerations located within the transition region are taken into account.

### 3.2. Wheel–Rail Impact Status

**Figure 6:**
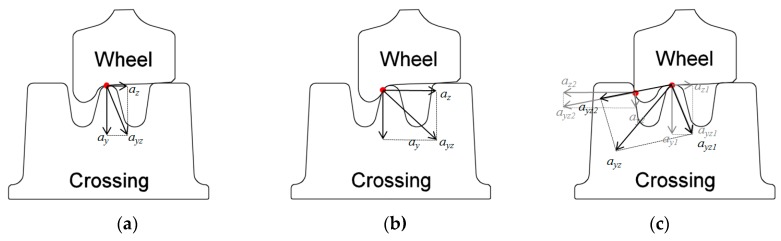
Wheel–rail contact situations: (**a**) regular contact; (**b**) irregular positive contact when wheel flange hits the crossing nose; (**c**) irregular negative contact when wheel flange hits the wing rail.

In an ideal situation, the wheel will pass through the transition region smoothly without flange contact (Figure 6a). In such a case, the vertical acceleration ($a_{y}$) will dominate the 3-D acceleration responses. However, in real life, due to disturbances existing in the track, each wheel passes the crossing at a different angle, which results in different impact accelerations in all the three directions. Referring to the measurement results, the impact angle can be defined by the factor of $k=a_{z}/a_{y}$. It has been found [1] that when an impact factor exceeds a certain level (|k|≥1), there is a large chance that the wheel flange will hit the nose rail or wing rail of the crossing (depending on the direction). Such flange contact is recognized as irregular positive (Figure 6b) or negative (Figure 6c) contact.

The irregular contact ratio is usually at a low level (below 3%) for well-maintained crossings, but might dramatically increase when damage occurs to the crossing (above 20%) [1]. Thus, the irregular contact ratio can be applied as a key indicator in assessing the conditions of railway crossings.

### 3.3. 3-D Acceleration Responses

**Figure 7:**
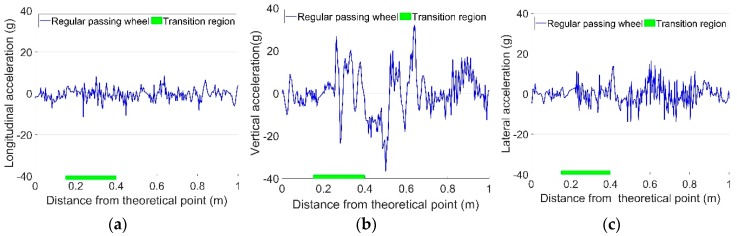
Examples of regular impact acceleration responses due to passing wheels. (**a**) Longitudinal acceleration; (**b**) Vertical acceleration; (**c**) Lateral acceleration.

**Figure 8:**
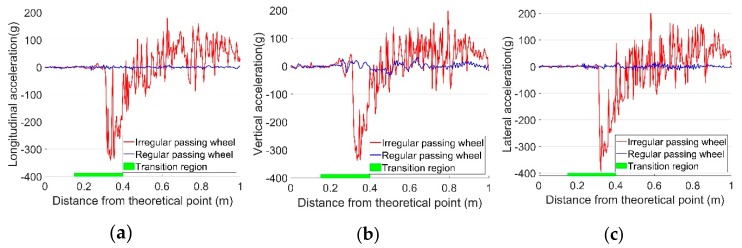
Examples of regular (same as Figure 7) and irregular impact acceleration responses due to passing wheels. (**a**) Longitudinal acceleration; (**b**) Vertical acceleration; (**c**) Lateral acceleration.

For the monitored crossing, the regular and irregular contact wheels showed dramatic differences in the 3-D impact acceleration responses (a). For regular passing wheels, the impact vertical acceleration was usually below 50 g, while such impact could be above 300 g for irregular passing wheels. Examples of the 3-D acceleration responses from typical regular and irregular passing wheels are shown in Figure 7 and Figure 8, respectively. In order to better understand the wheel–rail contact, the transition region obtained from field observation (Figure 5) is marked in the figures as a green line on the horizontal axis.

It can be seen from Figure 7 that $a_{y}$ is much higher than $a_{x}$ for a regular passing wheel, while $a_{z}$, meaning that the impact factor ($a_{z}/a_{y}$), is relatively small. It is also indicated that the wheel has two impacts on the crossing, with the first one (22 g) in the transition region and the second one (34 g) after the wheel load transit to the crossing nose rail. Even though the second impact has a higher amplitude, the first one is more damaging, since in the first impact location the nose rail is much thinner than in the second one.

For the irregular passing wheel presented in Figure 8, it can be seen that the impact was located in the transition region, and the accelerations in all three directions were very close to each other (in contrast to the regular passing wheel). Such a strong correlation of the acceleration responses reflects the intense wheel impact on the crossing nose rail and the rough transition of the wheel load from wing rail to the crossing nose rail. The big difference between the two typical wheel–rail impacts gives an example of the violent fluctuation of dynamic response results that can be observed in such crossings.

### 3.4. Impact Location and Fatigue Area

The impact location is defined as the point where the maximum wheel–rail impact occurs. As described previously, the impact location is restricted within the transition region. For the example given in Figure 4a, the impact location was 0.281 m from the TP.

The fatigue area is defined as the region where most of the wheel impacts are located and is calculated based on multiple wheel passages. In monitoring the conditions of railway crossings, the location and size of the fatigue area reflect the wheel load distribution along the crossing nose. In general, farther impact locations from the TP and wider fatigue areas indicate a better crossing condition.

**Figure 9:**
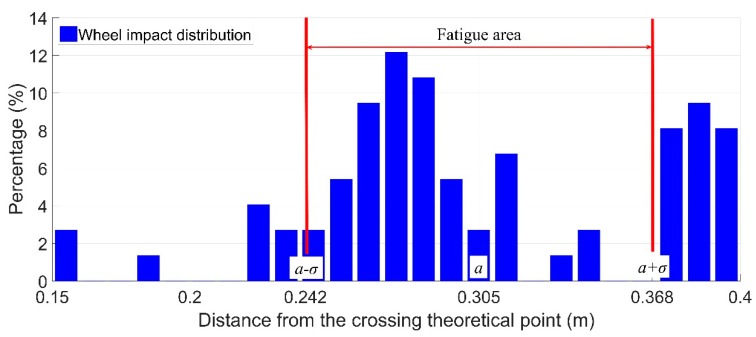
Example of fatigue area calculation.

In practice, to simplify the calculation procedure, the distribution of the wheel impacts due to multiple wheel passages is assumed to be normal distribution, the mean value a is the impact location and the confidence interval [a−σ,a+σ] is recognized as the fatigue area. An example of the fatigue area of the monitored crossing during a single day is given in Figure 9.

In this example, the wheel impact location was a=0.305 m, and the standard deviation of the simplified normal distribution was σ=0.063 m. Therefore, the fatigue area for the crossing during this monitoring day was between 0.242 and 0.368 m, with a size of 0.126 m. It can be noticed that the calculated fatigue area is not accurate, yet for condition monitoring in the long term, such simplification can provide reasonably acceptable results and highly improve the efficiency of data analysis.

### 3.5. Results from Multiple Monitoring Days

**Figure 10:**
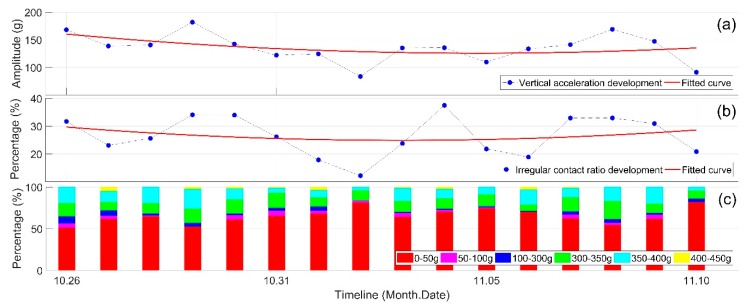
Development of the vertical acceleration responses in the monitored period. (**a**) Mean value of the vertical acceleration; (**b**) irregular contact ratio; (**c**) distribution of the acceleration responses for each day.

In order to describe the development of the crossing’s condition, the indicators are mainly used as statistical results over multiple monitoring days. An example of the development of vertical crossing acceleration responses as well as an irregular contact ratio is given for a span of 16 days in Figure 10. In this period, no track activities (e.g., maintenance) were performed, and the time frame was relatively too short for the condition of the crossing to degrade; therefore, the crossing condition can be assumed to be stable.

From Figure 10a it can be seen that the overall trend of the mean value of the accelerations is relatively stable, while the fluctuations of the responses are quite significant. The vertical accelerations have a minimum value of 84 g and a maximum value of 182 g. Such fluctuations resemble the fluctuations of the irregular contact ratio (Figure 10b). This resemblance will be further studied in the correlation analysis. It should be noted that the irregular contact ratio in the monitored period was above 10%, and for some days even it was higher than 30%, which is much higher than the previously studied 1:15 crossing [1] and reflects the abnormal condition of the monitored 1:9 crossing.

To summarize, the analyzed results have shown the following interesting features:

- a large difference in the dynamic responses from one passing wheel to another;
- a high irregular contact ratio due to multiple wheel passages during a single monitoring day; and
- highly fluctuating acceleration responses, as well as an irregular contact ratio during the short monitoring period.

All these features of the monitored 1:9 crossing indicate quite different performances from the previously studied 1:15 crossing. Investigating the sources of the fluctuation is necessary for a proper assessment of the crossing condition. Also, some condition indicators such as impact acceleration and the irregular contact ratio show possible correlations with each other. Figuring out the relationships between these indicators can help to reduce the amount of the required data, which will improve the efficiency of the post processing of the measurement results. These two questions can be investigated using correlation analysis, which will be presented in the next section. 

## 4. Correlation Analysis

As discussed in the previous section, a high fluctuation was observed in the vertical acceleration responses to the monitored crossing over a short period of time, and was unlikely to be related to structural changes. Considering that the interference factors from the train were minimized by data selection, one possible cause of the fluctuating dynamic responses might have been the continuously changing weather conditions.

### 4.1. Influence of the Weather

**Figure 11:**
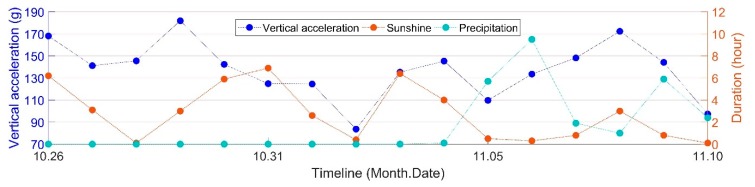
Development of vertical acceleration together with the durations of sunshine and precipitation.

It was discovered in the previous study [24] that temperature variation shows a good correlation with the acceleration fluctuation. In that study, the temperature fluctuation was considered to be the result of the duration of sunshine or precipitation. In order to assess the impact of the weather more accurately, the influences of weather conditions—including mean value of the daily temperature, daily sunshine and precipitation duration—will be analyzed. Figure 11 shows the fluctuation of crossing vertical acceleration responses with varying weather conditions.

From Figure 11 it can be seen that the fluctuating durations of sunshine showed a similar pattern to the crossing’s vertical acceleration responses. There seems to be connection between these two variables. For durations of precipitation, the connection with the vertical acceleration responses was lower. In order to quantitatively assess the impact of the weather, the correlations between the weather variables and condition indicators must be analyzed.

The weather data are obtained from the Royal Dutch Meteorological Institute (KNMI) [27] in days, and mainly consist of the following items:

- sunshine duration per day ($D_{s}$); and
- precipitation duration per day ($D_{p}$).

The crossing condition indicators were obtained from the crossing instrumentation, and the statistical results based on multiple monitoring days have been applied. The analyzed indicators include the following parts:

- longitudinal, vertical and lateral acceleration responses (a: $a_{x}$, $a_{y}$ and $a_{z}$, respectively);
- an irregular contact ratio ($I_{r}$); and
- wheel impact location ($L_{o}$) and size of the fatigue area ($F_{a}$).

### 4.2. Pearson’s Correlation Coefficient

In statistics, the linear correlation between two variables is normally measured using Pearson’s correlation coefficient *r*. For two variables X and Y with the same sample size of n, *r* can be obtained using the following formula:

(1) $$r_{X,Y}=\frac{\mathrm{cov}(X,Y)}{\sigma_{X}\sigma_{Y}}=\frac{E[(X-\mu_{X})(Y-\mu_{Y})]}{\sigma_{X}\sigma_{Y}}=\frac{1}{\sigma_{X}\sigma_{Y}}\cdot \frac{1}{n}\sum_{i=1}^{n}\left[(x_{i}-\mu_{X})(y_{i}-\mu_{Y}\right)]$$

(2) $$X=X(x_{1},x_{2},\ldots x_{n}),\;Y=Y(y_{1},y_{2},\ldots y_{n})$$

where

- cov(X,Y) is the covariance of *X* and *Y*;
- $\sigma_{X}\;\&\;\sigma_{Y}$ are the standard deviations of *X* & *Y*, respectively;
- $\mu_{X}\;\&\;\mu_{Y}$ are the mean values of *X* & *Y*, respectively; and
- E[…] is the expectation of the given variables

When *X* is in direct/inverse proportion to *Y*, then the correlation coefficient is

(3) $$r_{X,Y}=\frac{E[(X-\mu_{X})(Y-\mu_{Y})]}{\sigma_{X}\sigma_{Y}}=\pm \frac{\sigma_{X}\sigma_{Y}}{\sigma_{X}\sigma_{Y}}=\pm 1$$

If *X* and *Y* are independent, then the variable of $\left(x_{i}-\mu_{X})(y_{i}-\mu_{Y}\right)$ (1) could be a random positive or negative value. In case of a large amount of data (n→∞),

(4) $$\lim\frac{1}{n}\sum_{i=1}^{n}\left[(x_{i}-\mu_{X})(y_{i}-\mu_{Y}\right)]=0$$

Therefore, the value range of the correlation coefficient is $r_{X,Y}=[-1,1]$. $r_{X,Y}=\pm 1$ means that the two variables *X* and *Y* are perfectly correlated, and $r_{X,Y}=0$ means that *X* and *Y* have no correlation with each other. Otherwise, *X* and *Y* are considered partly correlated.

In different research fields, the gradation of the correlation index may have notable distinctions [28]. In some domains such as medicine and psychology, the requirement of the correlation coefficient—that a strong correlation is defined as |r|≥0.7—is relatively strict, while in other domains such as politics, |r|≥0.4 can already be considered a strong correlation. In this study, the structural responses and weather were indirectly associated. The three-level guideline modified from [29] is applied for the correlation strength analysis, as shown in Table 1.

### 4.3. Correlation Analysis

In the analysis presented here, the correlations between the dynamic responses of the crossing (a, $I_{r}$, $L_{o}$ and $F_{a}$) and the weather-related variables ($T_{m}$, $D_{s}$ and $D_{p}$) are studied. The data used for the correlation analysis are from 16 monitoring days (the same as in Figure 10, n = 16 in Equation (2)). The correlation within each group of parameters, as well as the cross-correlation between these two groups of parameters, will be analyzed.

The results are presented in Table 2. Nomenclature in the table is presented earlier in Section 4.1. The strong, moderate and weak correlation coefficients are marked with red, blue and black colors, respectively. The correlation results will be analyzed in the different categories presented below.

#### 4.3.1. Correlation of the Dynamic Responses

It can be seen from Table 2 it can be seen that the 3-D acceleration responses ($a_{x}$, $a_{y}$ and $a_{z}$) are very strongly correlated to each other. The irregular contact ratio ($I_{r}$) and the size of fatigue area ($F_{a}$) are also strongly correlated with a($a_{x}$, $a_{y}$ and $a_{z}$). It can be noted that the correlations between $F_{a}$ and a are negative, meaning that the increase of a is usually accompanied with the reduction of $F_{a}$. The correlations of the impact location ($L_{o}$) with other dynamic responses are not strong, meaning that $L_{o}$ is relatively independent from the other dynamic response. Some typical correlation results of the dynamic responses (framed in Table 2) are further discussed below.

**Figure 12:**
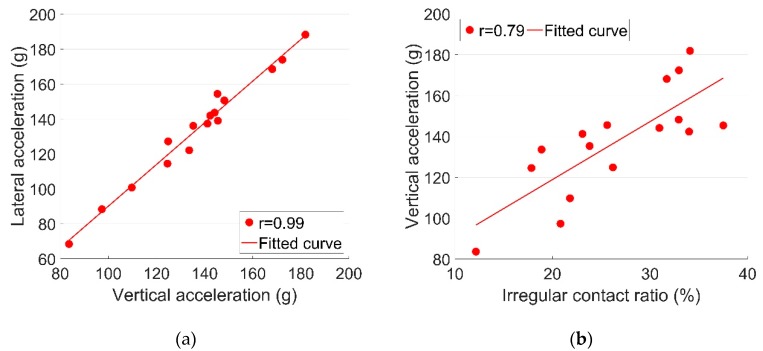
Correlations of the dynamic responses. (**a**) *a_y_-a_z_*; (**b**) *I_r_-a_y_*.

The very strong correlations of $a_{x}$, $a_{y}$ and $a_{z}$ (r≈1) indicate that the 3-D accelerations are synchronously developed. The correlation between $a_{y}$ and $a_{z}$ is demonstrated in Figure 12a. Therefore in practice, it is possible to use the accelerations only in one direction (e.g., $a_{y}$) to analyze the crossing behavior, which can help improve the efficiency in processing the measurement data.

The strong correlations between $I_{r}$ and a (Figure 12b) clearly indicate that the high acceleration responses are to a great extent contributed by the high ratio of irregular contact. This phenomenon could have been caused by temporary (not residual) rail displacements due to varying temperature forces in the rail. It has to be noted that all these responses ($I_{r}$ and a) fluctuated violently, a phenomenon that was likely caused by instable track conditions that were possibly affected by changes in weather conditions. This assumption will be verified later using a numerical model.

**Figure 13:**
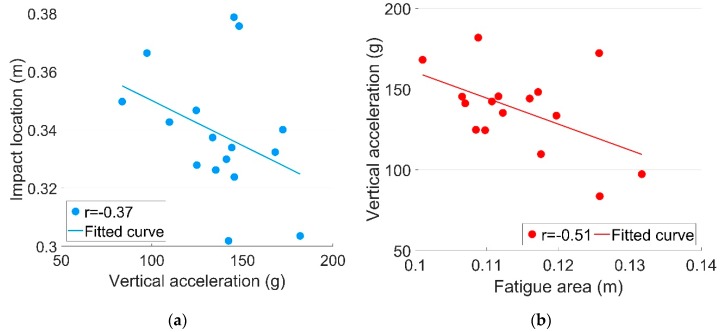
Correlations of the dynamic responses. (**a**) *a_y_-L_o_*; (**b**) *F_a_-a_y_*.

Figure 13 shows the correlation between $a_{y}$ and $L_{o}$. The negative result means that when a increased, there was a tendency for $L_{o}$ to be shifted closer to the crossing’s theoretical point, although the moderate correlation strength (r=−0.37) indicates that the connection between a and $L_{o}$ was rather limited. This might have been because the shift of $L_{o}$ was a long-term effect of rail geometry degradation [1]. However, the rail geometry was unlikely to be changed during the relatively short monitoring period (16 days), so the temporary change of a might not have directly resulted in the shift of $L_{o}$.

The correlation between $F_{a}$ and $a_{y}$ is shown in Figure 13b. Compared with $L_{o}$, $F_{a}$ was more likely to be reduced due to the increase of a. Combined with the strong correlation between a and $I_{r}$, it can be deduced that the impact locations of the irregular contact wheels tended to be centralized, while those of regular contact wheels were decentralized. Such a result confirms that a wider fatigue area will to some extent indicate a better crossing performance.

#### 4.3.2. Correlation of the Weather Conditions

**Figure 14:**
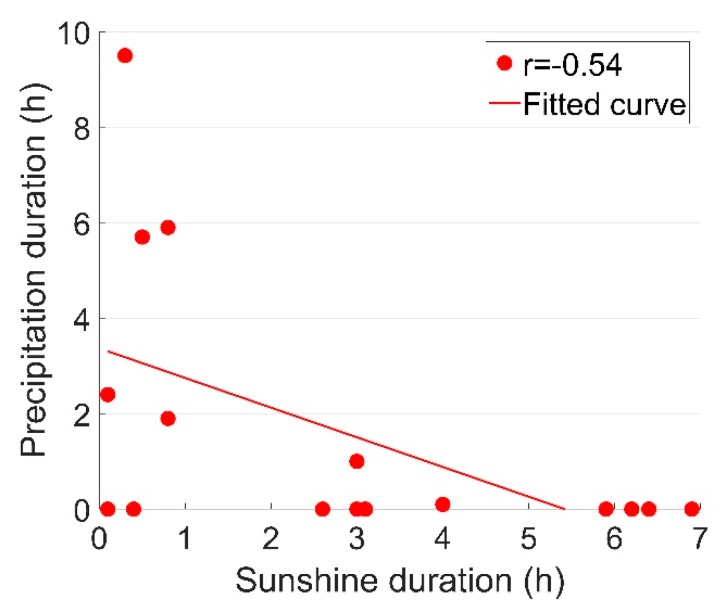
Correlation results between the sunshine and precipitation durations (*D_s_-D_p_*).

As can be seen from Table 2, the precipitation duration ($D_{p}$) had a strongly negative correlation with the sunshine duration ($D_{s}$), as shown in Figure 14. 

For the weather variables, $D_{s}$ and $D_{p}$ can be considered as two opposite weather conditions. From this point of view, the correlation coefficient of r=−0.54 is not very strong. Such results could be explained by the existence of cloudy/overcast conditions, and weather in a single day can switch among sun, rain and clouds/overcast. It can be noticed that in the monitored period, precipitation only occurred in 6 of the 16 days, which to some extent shows the complicity of the weather conditions.

#### 4.3.3. Cross-Correlation between Dynamic Responses and Weather Conditions

According to the correlation results presented in Table 2, the cross-correlations of $D_{p}$ with the dynamic responses are quite limited, except the moderate correlation with $F_{a}$. Meanwhile, $D_{s}$ was strongly correlated with $F_{a}$ and moderately correlated with all the other dynamic responses.

**Figure 15:**
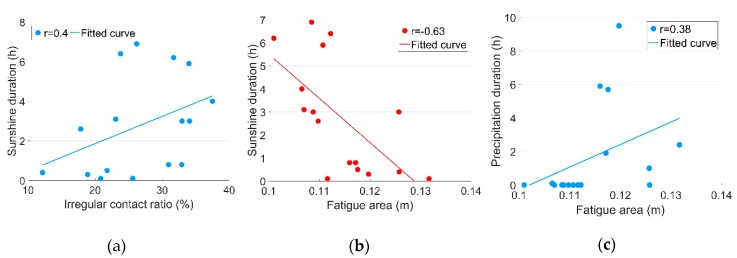
Cross-correlation results between the dynamic responses and weather conditions: (**a**) *I_r_*-*D_s_*; (**b**) *F_a_-D_s_*; (**c**) *F_a_-D_p_*.

The moderate correlation between $I_{r}$ and $D_{s}$ is shown in Figure 15a. Such a result can be explained by the fact that an increase in rail temperature due to sunshine causes the displacements in the turnout. Due to these geometrical changes, the wheel cannot pass the crossing normally anymore and results in the increase of the irregular contact. Such a result is consistent with the moderate correlations between $D_{s}$ and a.

The correlation of $D_{s}$ with $F_{a}$ was stronger than with the other dynamic responses (r=−0.63, Figure 15b), meaning that sunshine-initiated rail displacements were likely to occur primarily in centralized impact locations, which may have increased the likelihood of irregular contact.

**Figure 16:**
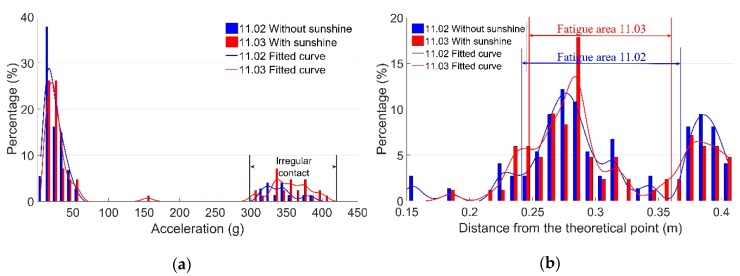
Influence of sunshine on the dynamic responses. (**a**) Vertical acceleration distribution; (**b**) fatigue area analysis.

An example for demonstrating the influence of sunshine on the dynamic responses of the monitored crossing is given in Figure 16. In this example, there was hardly any sunshine on one day (11.02), and a long period of sunshine on another day (11.03) (Figure 11). It can be seen that on 11.03 (with sunshine), $I_{r}$ was higher (Figure 16a) and $F_{a}$ was slightly narrower (Figure 16b). Such results indicate that the temporary effect of sunshine can lead to changes in the crossing performance.

**Figure 17:**
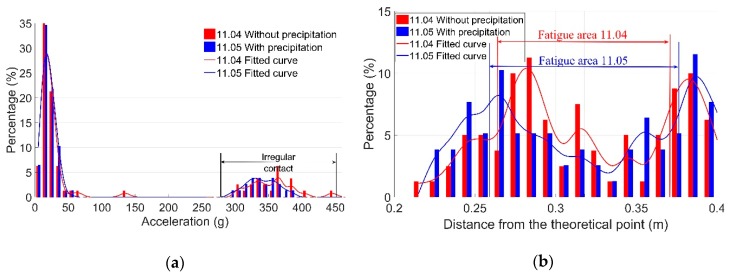
Influence of precipitation on the dynamic responses. (**a**) Vertical acceleration distribution; (**b**) fatigue area analysis.

The moderate correlation between $D_{p}$ and $F_{a}$ is shown in Figure 15c. Considering that the correlations between $D_{p}$ and $D_{s}$ were not very strong, the moderate correlation between the dynamic responses and weather conditions can already indicate a measure of impact. An example of the measured dynamic responses of the crossing for a day without precipitation (11.04) and a day with precipitation (11.05, Figure 11) are shown in Figure 17.

It can be seen in Figure 17 that on the day with precipitation (11.05), $I_{r}$ was slightly lower than on the day without precipitation (11.04), and $F_{a}$ was wider. The reason for such results could be that precipitation may reduce the friction coefficient on the rail’s surface and make the transition of the wheel load smoother. This assumption will be verified using a numerical model in the next section.

It should be mentioned that the subgrade of the monitored crossing was relatively soft, with canals on both sides of the track. Persistent precipitation could change the property of the subgrade and further affect the dynamic performance of the track. Therefore, the influence of precipitation can be quite complicated.

Based on the correlation analysis, the main conclusions can be drawn as follows:

- The accelerations in all three directions developed synchronously. In monitoring crossing conditions, it is sufficient to use vertical acceleration to represent the 3-D acceleration responses. Through this, the data processing procedure can be simplified.
- The strong correlation between $I_{r}$ and $a_{y}$ indicates that irregular contact is likely to result in high-impact accelerations. Such a result confirms that $I_{r}$ can be used as an indicator for assessing crossing conditions. A high value of $I_{r}$ indicates a degraded condition of the monitored crossing.
- The high (moderate/strong) correlation results between $D_{s}$ and the dynamic responses of the crossing clearly indicate the influence of weather. It can be concluded that significant fluctuations in accelerations during a relatively short period are caused by changes in weather conditions. To verify this, a numerical model will be used in the next section.

## 5. Numerical Verification

In general, solar radiation is one of the major sources of rail thermal force. Depending on the sunshine duration, the associated rail temperature can rise to 40 °C higher than the ambient air temperature [30]. The change in rail temperature will increase the rail stress and amplify lateral displacements in the rail. The lateral displacements will then increase the uncertainty of the impact angle of a wheel in the railway crossing, eventually leading to an increase in the acceleration responses of some passing wheels, as shown in Figure 10. 

Precipitation will introduce water to the rail surface that acts as a lubrication layer, which will reduce the friction coefficient in the wheel–rail interface [21]. It has been studied [31] that a low friction coefficient can be helpful in reducing hunting oscillation and, in contrast to sunshine, can reduce the impact angle of a wheel in the railway crossing.

The above-mentioned effects of temperature and friction variation corresponding to sunshine and precipitation are implemented in the multi-body system (MBS) model described below.

### 5.1. MBS Model Setup and Validation

**Figure 18:**
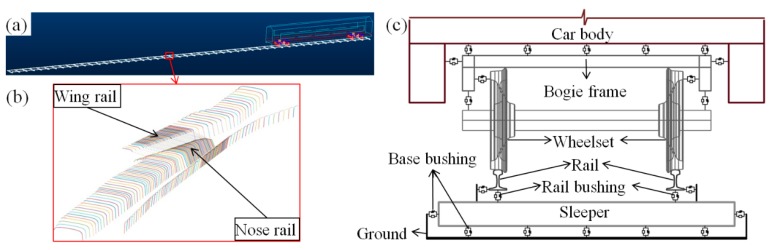
MBS model. (**a**) Vehicle-track model; (**b**) crossing profiles; (**c**) flexible connections in the model.

In order to verify the weather effect hypotheses, a model for analyzing vehicle-crossing interaction developed according to the MBS method (implemented in VI-Rail software) will be used, as shown in Figure 18a. The track model is a straight line with a crossing panel (Figure 18b) situated in the middle of the track. The total length of the track model is 100 m, which allows enough preloading time for the vehicle before it enters into the crossing panel, as well as enough space after the vehicle passes through the crossing. The crossing geometry is defined by the control cross-sections, and the profiles between two pre-defined cross-sections are automatically interpolated using the third-order spline curve. In the track model, the rail is considered to be lumped masses on the sleepers connected with a massless beam. The flexible layers under the rail are the rail bushing that represents the rail pads and clips, and the base busing representing the ballast bed (Figure 18c).

The crossing model is the same as the monitored 1:9 casted manganese crossing with a rail type of UIC54 E1. The track parameters of Dutch railways [32] applied in the model are shown in Table 3.

The vehicle model was developed based on a VIRM locomotive with a total length of 27.5 m comprising a car body, front bogie and rear bogie. In the vehicle model, the car body and bogie frames, as well as the wheel sets, are modelled as rigid bodies with both primary and secondary suspensions taken into account (Figure 18c) [33]. The vehicle travels with a velocity of 140 km/h, the same as in the data analysis measurements. The wheels use a S1002 profile with a wheel load of 10 t. The wheel–rail contact model is defined as the general contact element and uses actual wheel and rail profiles as an input, which allows variable wheel and rail profiles.

**Figure 19:**
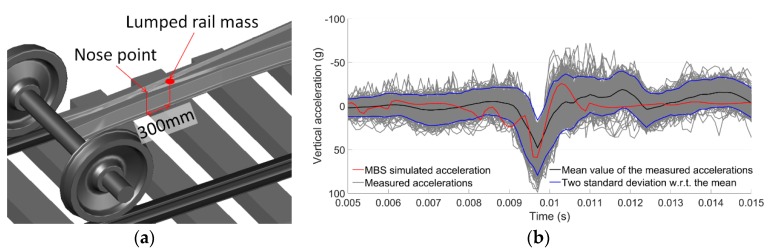
MBS model validation: (**a**) rail element for acceleration extraction; (**b**) comparison of MBS simulated acceleration with the measured ones in a time domain.

The MBS vehicle–track model was validated using the measured acceleration responses from the crossing with the same design and stable conditions. Since the validation simulation was based on ideal track conditions, only the acceleration responses with regular wheel–rail contact were used in the comparison. The selected element for acceleration extraction was the rail with lumped mass (Figure 19a) from the same location as the instrumented accelerometer (Figure 2).

The validation results are shown in Figure 19b. It can be seen that the simulation results (red line) are quite comparable with the measured accelerations (black line). The magnitude of the simulated vertical acceleration during impact was around 55 g, which is comparable with the mean value of the measured acceleration responses (47 g). Although tolerable deviations of the impact signals exist, the simulation results agree reasonably with the measurements. It can be concluded that the MBS model can catch the main features of the wheel–rail impact during crossing and can be used to analyze crossing performance. Further details about the numerical model development and validation can be found in [34].

### 5.2. Numerical Analysis

#### 5.2.1. Effect of Sunshine

**Figure 20:**
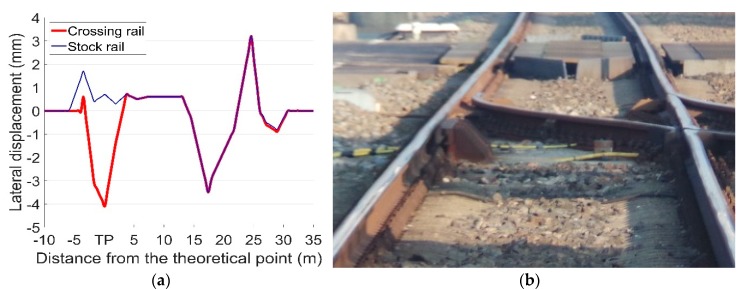
(**a**) Temperature-initiated rail lateral displacement in FE simulation (adapted from Figure 11.15 in [23]); (**b**) the monitored crossing.

In the previous study [35], the displacements of a turnout due to the change of the rail temperature were analyzed using a finite element (FE) model. The simulation results indicated that when the rail temperature was increased (from a stress-free temperature) by 40 °C, the turnout rails were laterally displaced up to 4 mm, as shown in Figure 20a. These results are applied in the MBS vehicle-crossing model as the sunshine-initiated lateral displacements. It should be noted that this simulation is based on ideal track conditions. In the case of a degraded track, the temperature-initiated lateral displacements could be amplified.

In order to take the track degradation into account for the degraded track condition, the input lateral rail displacements in the MBS model are assumed to be twice as high as the ideal track condition (with maximum lateral rail displacements of 8 mm). The effect of precipitation is not taken into account and the friction coefficient of f=0.4 is used. Based on the above assumptions, the vertical accelerations and transition regions of the rail are simulated and presented below.

**Figure 21:**
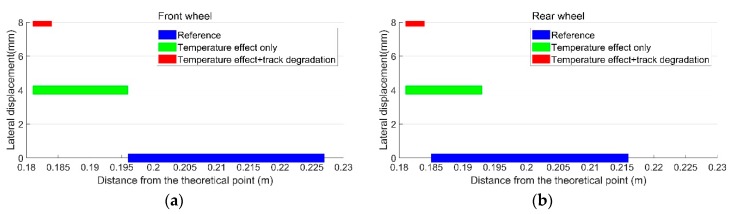
Transition regions of the crossing. (**a**) Front wheel; (**b**) rear wheel.

The calculated transition regions under different track conditions are shown in Figure 21. In the reference condition with no lateral displacement in the track, the sizes of the transition regions for the front wheel and the rear wheel are both 0.031 m [34]. When the temperature-initiated track displacements are taken into account, the transition regions shift closer to the theoretical point and the sizes reduce dramatically to 0.015 m for the front wheel and 0.012 m for the rear wheel. For the degraded track with higher rail displacements, the size of the transition region is only 0.004 m.

**Figure 22:**
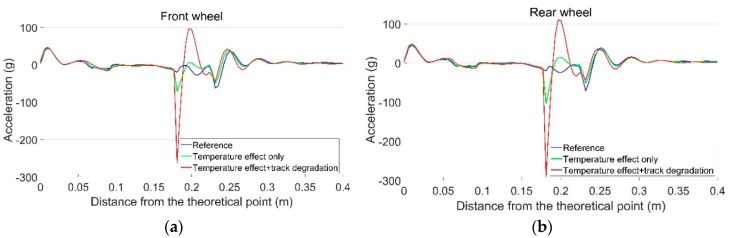
Vertical rail accelerations due to passing wheels. (**a**) Front wheel; (**b**) rear wheel.

The vertical acceleration response of the rail due to passing wheels is shown in Figure 22. It can be seen that lateral displacement in the rail can result in higher acceleration responses caused by both the front and rear wheels. Combined with the results of the transition region (Figure 21), the simulation results confirm the correlation results (Figure 15a,b) that the long sunshine duration, which will result in a higher temperature in the rail, can lead to a centralized impact location and higher impact acceleration responses at the crossing.

It can be also seen that with the existence of rail displacement, the acceleration response caused by the rear wheel is higher than that caused by the front wheel from the same bogie. These results indicate that the performance of the rear wheel is not only affected by rail displacement, but also by the passing condition of the front wheel.

In case of a degraded track, higher rail displacements may lead to much higher acceleration responses as a result of both the front and rear wheels. Such impact accelerations (near 300 g) are close to the amplitude of the acceleration responses due to the irregular impacts in the measurements (Figure 16a and Figure 17a). The simulation results prove that the lateral rail displacements caused by increases in rail temperature, in combination with track geometry deviations, can result in high wheel–rail impact accelerations.

#### 5.2.2. Effect of Precipitation

**Figure 23:**
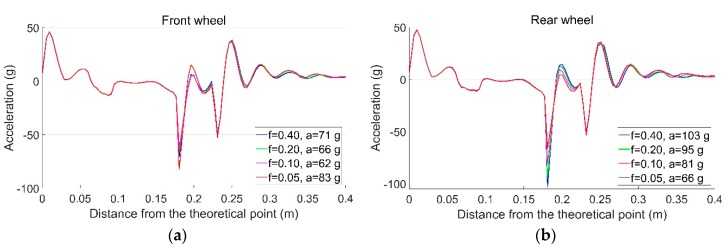
Vertical accelerations of the rail due to the passing wheels. (**a**) Front wheel; (**b**) rear wheel.

With the influence of precipitation, the friction coefficient (f) in the wheel–rail interface can vary from 0.4 to 0.05 [35]. In this study, the precipitation effect is simulated by a reduction of f. The temperature-initiated rail displacements under ideal track conditions are taken into account. Calculations of rail accelerations resulting from passing wheels are shown in Figure 23.

For the front wheel, when f is reduced from 0.4 to 0.1, the impact acceleration gradually reduces from 71 to 62 g. However, when f=0.05, the maximum impact acceleration is increased to 83 g. Such results show that reducing the friction coefficient is not always helpful for the dynamic performance of the crossing. For the rear wheel, the reduction of f results in a decreased impact acceleration from 103 to 66 g. As discussed previously, the high rail acceleration responses due to the rear wheel are affected by the movement of the front wheel. In this case, the lowered f can help the wheelset return to a balanced position faster due to lower lateral restraint, which reduces the influence of the front wheelset on the rear wheelset from the same bogie.

It can be concluded that the change of f due to precipitation has an influence on the dynamic performance of the crossing, but the effect of a lower f is not always positive. Such results prove the correlation results indicating that an increase of $D_{p}$ tends to result in lower acceleration responses, but the correlation strength is not high. The moderate correlation between $D_{p}$ and $F_{a}$ is also consistent with the simulation results that each wheel passes through the crossing more independently, which leads to less centralized impact locations.

### 5.3. Discussion

In this section, the MBS model for vehicle–crossing interaction analysis was briefly introduced. Using this model, the sunshine and precipitation effects were simulated as rail displacements and reduced f in the wheel–rail interface, respectively. The simulation results indicate that the rail displacements due to sunshine can lead to an increase in wheel-crossing impact acceleration. Combined with track degradation, such an effect could be highly amplified. Meanwhile, a lower f in the wheel–rail interface due to precipitation might reduce the interaction effect of two wheelsets from the same bogie, but cannot help improve track conditions. Combined with the measurement results, it can be concluded that the monitored crossing was not in the ideal condition, and possessed a certain degree of track degradation that made it more sensitive to changes in weather conditions.

## 6. Conclusions and Future Work

### 6.1. Conclusions

In this study, the conditions of a railway crossing were monitored, and the results were presented. The indicators for assessing the conditions of a crossing were briefly introduced. Inspired by the observed connection between vertical acceleration responses of the crossing and variations in the sunshine duration, correlations of the dynamic responses and weather conditions were calculated. Using the vehicle-crossing MBS model, the influence of weather on the performance of the crossing was verified. The main conclusions of this study can be drawn as follows:

- The strong correlations between the dynamic responses show that the measurement results can be simplified and the crossing conditions can be assessed by only a few indicators (e.g., vertical acceleration, irregular contact ratio and fatigue area).
- The correlation results between the dynamic responses of the crossing and sunshine duration explain the fluctuation of dynamic responses over a short period of time. Such results confirm the temporary influence of weather on the performance of a crossing.
- The correlation results between sunshine duration and precipitation duration, as well as between precipitation duration and the dynamic responses of the crossing, indicate the complexity of the effect of precipitation.
- The simulation results not only verify the impact of weather on the dynamic performance of the crossing, but also indicate that the condition of the track at the monitored crossing was degraded. In cases of track degradation, the influence of weather can be amplified.

In monitoring the conditions of railway crossings, the correlation results among dynamic responses can be used to simplify measurement data. The verified weather effects explain the fluctuation of the dynamic responses over a short time period, which provides the basis for the measurement data regression. It should be noted that although sunshine variation is a short-term effect, the interaction of sunshine with the degraded track can turn this temporary interruption into a permanent track deformation, which will further accelerate the degradation of the track. In monitoring the conditions of railway crossings, the influence of weather can be eliminated through data regression to describe the structural degradation procedure, but the reflected track problem has to draw enough attention. Ensuring good track condition will not only help prolong service life of the crossing, but will also reduce the influence of varying weather conditions.

### 6.2. Future Work

This study was based on monitoring the conditions of railway crossings. It can be imagined that weather variation might also have an impact on other track sections, especially vulnerable parts such as transition zones, insulated joints, sharp curves, and so on. In the future, the effects of weather on other parts can be further investigated, which will improve the universality of this study and provide broader information for railway track management.

## Figures and Tables

**Table 1:** The three-level correlation strength guideline.

r	Correlation Strength
|r|<0.3	Weak
0.3≤|r|<0.5	Moderate
0.5≤|r|<1	Strong

**Table 2:** Correlation coefficients for dynamic responses and weather variables.

r	*a_x_*	*a_y_*	*a_z_*	*I_r_*	*L_o_*	*F_a_*	*D_s_*	*D_p_*
***a_x_***	1	0.98	0.99	0.84	−0.30	−0.56	0.43	−0.23
***a_y_***		1	0.99	0.79	−0.37	−0.51	0.36	−0.17
***a_z_***			1	0.85	−0.32	−0.53	0.42	−0.22
***I_r_***				1	−0.09	−0.42	0.40	−0.22
***L_o_***					1	0.36	−0.39	0.14
***F_a_***						1	−0.63	0.38
***D_s_***							1	−0.54
***D_p_***								1

**Table 3:** Track parameters applied in the MBS model.

Track Components		Stiffness, MN/m	Damping, kN·s/m
Rail pad/Clips	Vertical	1300	45
	Lateral	280	580
	Roll	360	390
Ballast		45	32
